# Supplementary material for: Defence systems drive accessory genome interactions in Pseudomonas aeruginosa
Source: ISME Commun. 2026 Jun 1;6(1):ycag130. doi: 10.1093/ismeco/ycag130 (PMC13452453; doi:10.1093/ismeco/ycag130)
Supplement: Supplementary file 1 [file supplementary_material_ycag130.zip › Supplementary_Figure_1_ycag130.pdf]

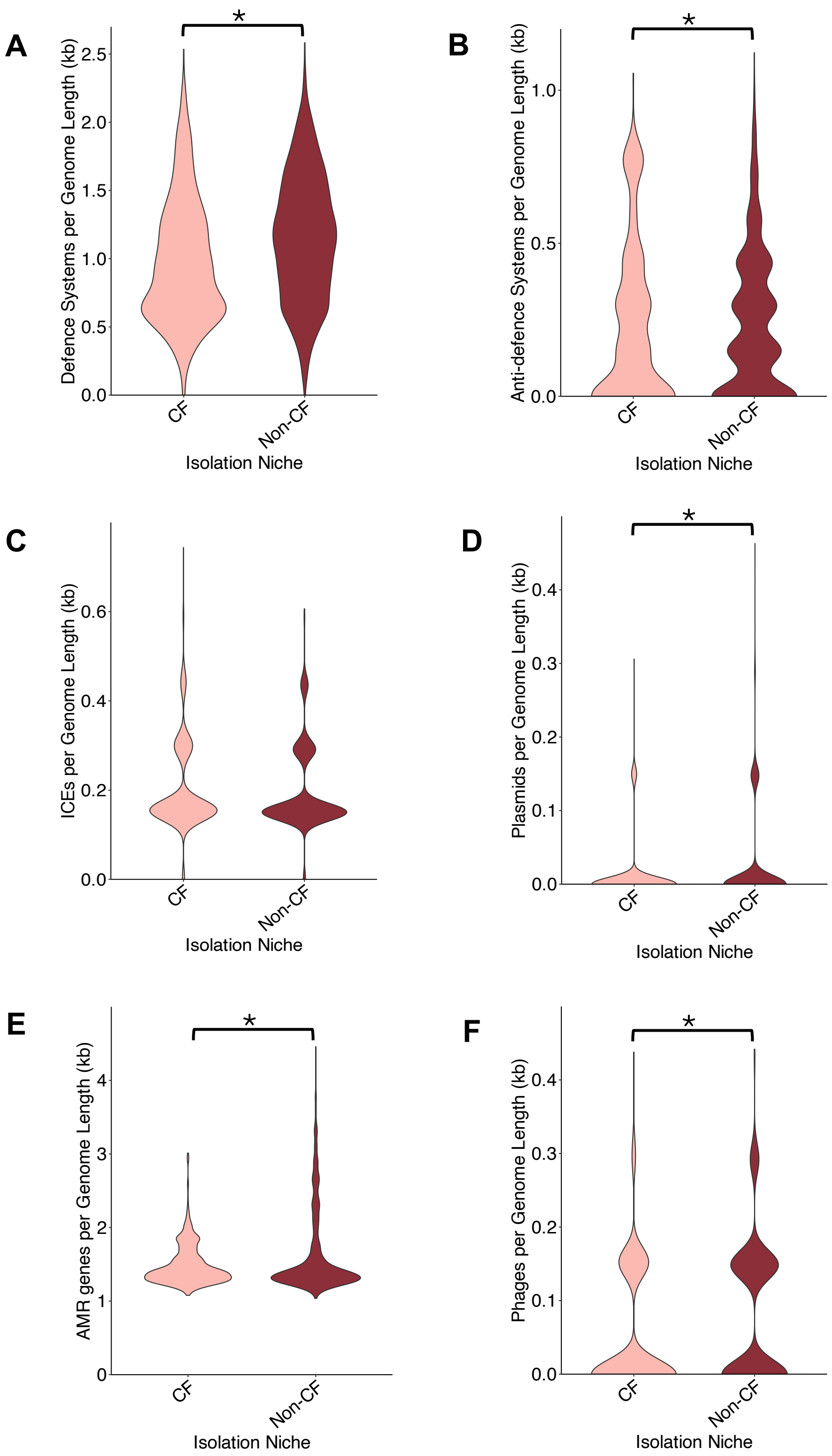

Supplementary Figure 1. Number of defence systems **(A)**, anti-defence systems **(B)**, ICEs **(C)**, plasmids **(D)**, ARGs **(E)** and phage **(F)** present in *P. aeruginosa* per genome length

(kb) of genomes isolated from CF patients and non-CF sources. Significant differences in number of accessory genome elements per genome length (kb) between genomes isolates from CF and non-CF niches is denoted with a * (*t*-test $p<0.05$). ICE = Integrated Conjugative Element ARGs = Antimicrobial Resistance Genes CF = cystic fibrosis DS = Defence System.
