# Supplementary material for: Defence systems drive accessory genome interactions in Pseudomonas aeruginosa
Source: ISME Commun. 2026 Jun 1;6(1):ycag130. doi: 10.1093/ismeco/ycag130 (PMC13452453; doi:10.1093/ismeco/ycag130)
Supplement: Supplementary file 1 [file supplementary_material_ycag130.zip › Supplementary_Figure_2_ycag130.pdf]

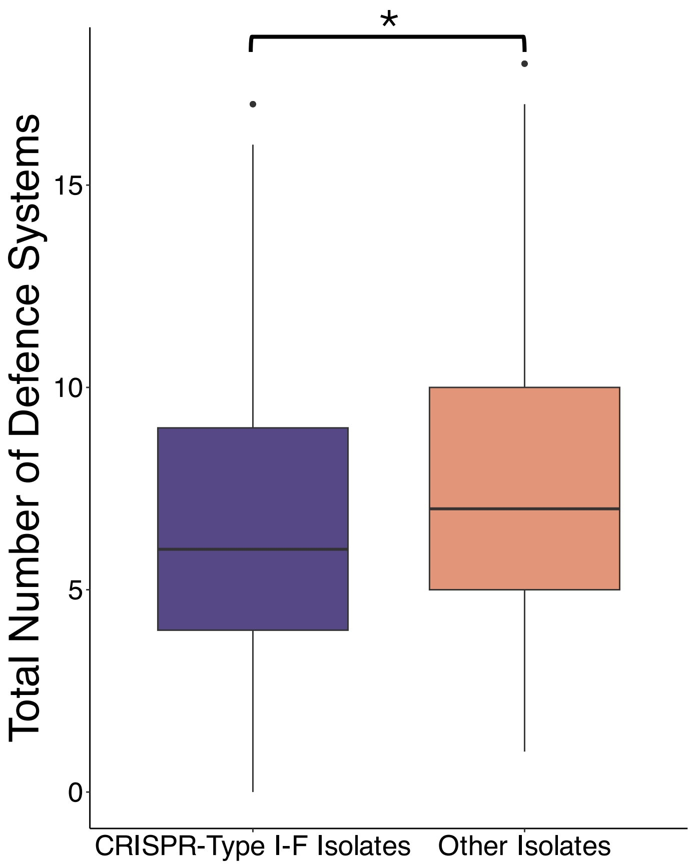

To evaluate the possible dominant negative association of CRISPR-Cas Type I-F, we determined that those genomes in this study containing CRISPR-Cas Type I-F (n=159/4288) contained fewer DSs 6.7 (range 0-17), compared with 7.2 (range 1-18) in non-CRISPR-Cas Type I-F containing genomes (t-test, $p<0.05$).

Supplementary Figure 2. Comparison of the number of DSs found in *P. aeruginosa* isolates containing the DS CRISPR-Type I-F (n=1591) and isolates that did not (n=2683). The plot shows the median (line), interquartile ranges (box), and range (whiskers) of the number for each group. Outliers are shown as individual markers. The asterisk indicates a statistically significant difference ($p<0.05$) as measured by a *t*-test.
