# Supplementary material for: Defence systems drive accessory genome interactions in Pseudomonas aeruginosa
Source: ISME Commun. 2026 Jun 1;6(1):ycag130. doi: 10.1093/ismeco/ycag130 (PMC13452453; doi:10.1093/ismeco/ycag130)
Supplement: Supplementary file 1 [file supplementary_material_ycag130.zip › Supplementary_Figure_3_ycag130.pdf]

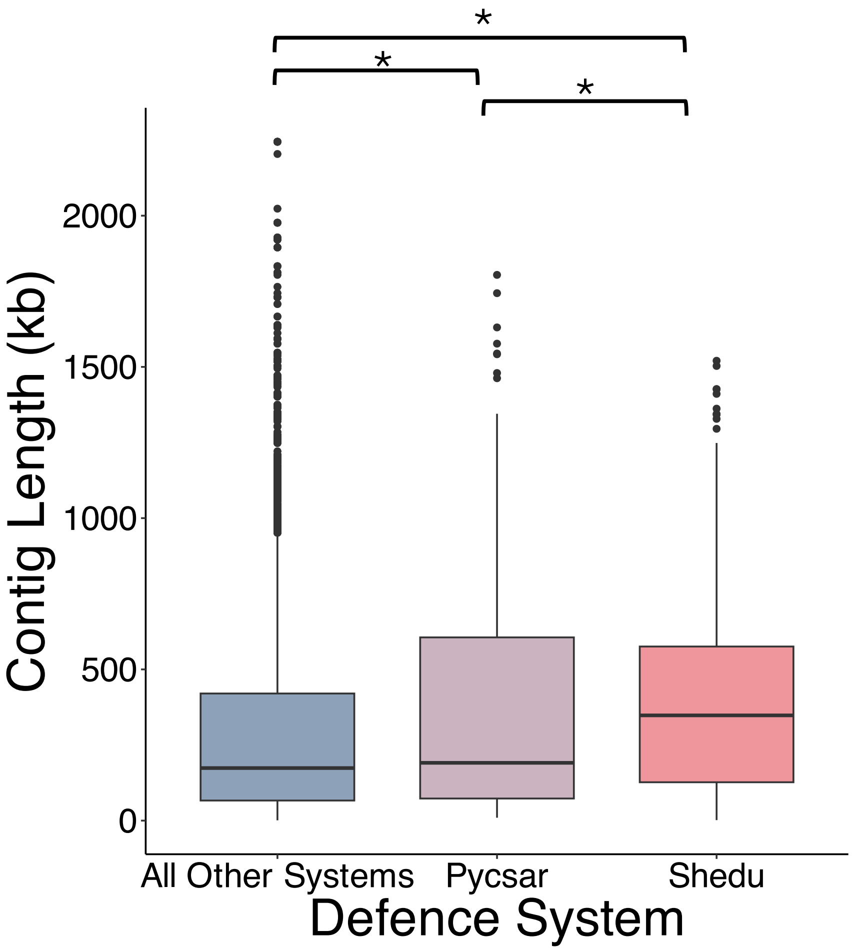

Shedu and Pycsar, which were on average found to co-localise in lower proportions, compared to other non-Pycsar/Shedu containing pairs (average proportion of co-localisation in DS pairs containing, Pycsar = 0.01, Shedu = 0.06 and all other system pairs = 0.2. To confirm that these apparently low-rates of non-co-localisation of Pycsar and Shedu were not the result of disproportionately shorter contigs, we assessed the average contig length (kb) of sequences containing these DSs relative to other DS-containing contigs. This revealed that Shedu or Pycsar containing contigs (average of 393 kb and 349 kb respectively) were slightly longer than contigs containing other DSs (average of 275 kb) (Kruksall-Wallis rank sum test and Dunn's post hoc test = p<0.05) suggesting that this is a genuine interaction between Shedu and Pycsar, and other systems and might represent either functional redundancy or mechanistic antagonism with other systems.

Supplementary Figure 3. Comparison of length in kilobases of contiguous sequences containing either Pycsar (n=539), Shedu (n=586) or all other DS (n=23471). The plot shows the median (line), interquartile ranges (box), and range (whiskers) of the number

for each group. Outliers are shown as individual markers. The asterisk indicates a statistically significant difference (p<0.05) as measured by a Kruksall-Wallis rank test followed by a Dunn's post hoc test for pairwise comparisons
